# The metabolomics of asthma control: a promising link between genetics and disease

**DOI:** 10.1002/iid3.61

**Published:** 2015-05-07

**Authors:** Michael J McGeachie, Amber Dahlin, Weiliang Qiu, Damien C Croteau-Chonka, Jessica Savage, Ann Chen Wu, Emily S Wan, Joanne E Sordillo, Amal Al-Garawi, Fernando D Martinez, Robert C Strunk, Robert F Lemanske, Andrew H Liu, Benjamin A Raby, Scott Weiss, Clary B Clish, Jessica A Lasky-Su

**Affiliations:** 1 Department of Medicine, Channing Division of Network Medicine, Brigham and Women's Hospital and Harvard Medical SchoolBoston, Massachusetts, USA; 2 Children's Hospital and Harvard Medical SchoolBoston, Massachusetts, USA; 3 Department of Population Medicine, Harvard Medical School and Harvard Pilgrim Health Care InstituteBoston, Massachusetts, USA; 4 Arizona Respiratory Center and BIO5 Institute, University of ArizonaTucson, Arizona, USA; 5 Department of Pediatrics, Division of Allergy, Immunology and Pulmonary Medicine, Washington University School of MedicineSt. Louis, Missouri, USA; 6 University of Wisconsin School of Medicine and Public HealthMadison, Wisconsin, USA; 7 Department of Pediatrics, Division of Allergy and Clinical Immunology, National Jewish Health and University of Colorado School of MedicineDenver, Colorado, USA; 8 Broad InstituteCambridge, Massachusetts, USA

**Keywords:** Albuterol, asthma, epigenetics, genetics, metabolomics

## Abstract

Short-acting β agonists (e.g., albuterol) are the most commonly used medications for asthma, a disease that affects over 300 million people in the world. Metabolomic profiling of asthmatics taking β agonists presents a new and promising resource for identifying the molecular determinants of asthma control. The objective is to identify novel genetic and biochemical predictors of asthma control using an integrative “omics” approach. We generated lipidomic data by liquid chromatography tandem mass spectrometry (LC-MS), ­ using plasma samples from 20 individuals with asthma. The outcome of interest was a binary indicator of asthma control defined by the use of albuterol inhalers in the preceding week. We integrated metabolomic data with genome-wide genotype, gene expression, and methylation data of this cohort to identify genomic and molecular indicators of asthma control. A Conditional Gaussian Bayesian Network (CGBN) was generated using the strongest predictors from each of these analyses. Integrative and metabolic pathway over-representation analyses (ORA) identified enrichment of known biological pathways within the strongest molecular determinants. Of the 64 metabolites measured, 32 had known identities. The CGBN model based on four SNPs (rs9522789, rs7147228, rs2701423, rs759582) and two metabolites—monoHETE_0863 and sphingosine-1-phosphate (S1P) could predict asthma control with an AUC of 95%. Integrative ORA identified 17 significantly enriched pathways related to cellular immune response, interferon signaling, and cytokine-related signaling, for which arachidonic acid, PGE2 and S1P, in addition to six genes (CHN1, PRKCE, GNA12, OASL, OAS1, and IFIT3) appeared to drive the pathway results. Of these predictors, S1P, GNA12, and PRKCE were enriched in the results from integrative and metabolic ORAs. Through an integrative analysis of metabolomic, genomic, and methylation data from a small cohort of asthmatics, we implicate altered metabolic pathways, related to sphingolipid metabolism, in asthma control. These results provide insight into the pathophysiology of asthma control.

## Introduction

Asthma affects over 300 million individuals in the world 1, and the primary reliever medications used in asthma are short-acting β_2_ agonists (SABA, e.g., albuterol) which relax bronchial smooth muscle by activating β_2-_adrenergic receptors. Use of SABA is a recognized marker for uncontrolled asthma 2, which leads to asthma exacerbations, the most common health-related cause of lost school and work days and contributes to the more than $50 billion spent annually on asthma 3,4. Asthma is a complex disease with both environmental and genetic components 5,6. Although a number of genetic determinants have been identified 7, much remains to be understood about how these variants impact the maintenance and control of the disease, and how these can lead to exacerbations. Metabolomics, the systematic analysis of small molecules, has been successfully used to identify new disease biomarkers 8–10. Metabolomics provides an integrated profile of biological status reflecting the net results of genetic and environmental interactions 11,12, thereby making this a promising new strategy to examine in the context of asthma control, an approach that has already been advocated in the literature 13. In contrast to transcriptional, translational, and post-translational changes, metabolites have the distinct advantage of being more proximal markers of disease processes. Metabolic profiling can also capture the history of past exposures such as hypermethylation and response to hypoxia, making this approach particularly relevant to asthma 14. Therefore, metabolomic analysis offers a novel approach for understanding asthma control.

To date, few metabolomics studies have been performed with asthma 14–21. Furthermore, none of these studies utilized other forms of genomic data to inform their findings. The objective of this study was to identify novel predictors of asthma control using an integrative “omics” approach that integrates genotype, expression, metabolomics, and methylation profiling data.

## Materials and Methods

### Study population

The Asthma BioRepository for Integrative Genomic Exploration (Asthma BRIDGE) is an open-access collection of cDNA and DNA from primary asthma-relevant cell types and immortalized cell lines from more than 1,450 well-characterized subjects participating in ongoing genetic studies of asthma, and an accompanying database of phenotype, genome-wide SNP genotype, gene expression, and methylation data. For this analysis, we obtained samples for 20 individuals from the Childhood Asthma Research and Education (CARE) Network cohort, a subset of Asthma BRIDGE 22. The CARE Network enrolled children aged 1–18 years with a confirmed diagnosis of asthma or wheezing illness 23. Details relating to the study protocols were previously published 22. We stratified our cohort into patients with uncontrolled and controlled asthma based on self-reported use of SABA inhalers in the week preceding blood draw for Asthma BRIDGE genomic assays, as an indicator of asthma control.

### Data

#### Metabolomic profiling

We used liquid chromatography tandem mass spectrometry (LC-MS) to measure lipid metabolites. Quadrupole orbitrap mass spectrometers (two Q Exactive and one Exactive Plus MS, Thermo Scientific) enabled the measurement of high-resolution metabolites of both known and unknown identities in the same experiment. Targeted datasets contained lists of metabolites of known identity and the integrated LC-MS peak areas measured in each sample. Targeted datasets contained a large number of de-isotoped LC-MS peaks per sample that were indexed by mass to charge ratio and retention time. A subset of these signals was readily identified by comparison to reference standards and reference samples. Lipids were extracted from plasma (10 μL) using 19 volumes of 100% isopropanol. Extracts were separated using reversed phase chromatography and full scan MS data were acquired in the positive ion mode. A total of 68 metabolites were generated, of which two had no variation, and another two had missing values for more than half the subjects. These metabolites were removed before further analysis, leaving 64 metabolites successfully assayed. Principal component analysis (PCA) identified four subjects as possible outliers; these were included in subsequent analyses when appropriate.

#### Genome-wide gene expression profiling

Existing data were obtained through Asthma BRIDGE. Stimulated CD4+ T-cell lymphocyte RNA samples from CARE were previously processed as part of a group of 2,317 samples of diverse tissues from within Asthma BRIDGE as follows. The samples were assayed for 47,036 different transcripts using Illumina Human HT-12 v4 arrays, as per manufacturer's protocol (Illumina, Inc., San Diego, CA). After removing 27 outlier probes, the expression values of the remaining 47,009 probes were log_2_-transformed and quantile-normalized. Among the 2,068 non-duplicate Asthma BRIDGE samples with good signal-to-noise ratios (p95/p05>6), 16 were CARE samples that had also been successfully assayed for each of 64 metabolites. PCA of gene expression identified two CARE outliers, which were removed from related downstream analyses.

We tested the association between each metabolite with each of the 47,009 mRNA probes, adjusted for age and gender, using the *limma* Bioconductor package for the R programming language 24. Association findings that were due to high leverage points were removed. Analyses were adjusted for multiple testing using the False Discovery rate (FDR) 25.

#### Genome-wide methylation profiling

Existing data were obtained through Asthma BRIDGE. Genome-wide DNA methylation data was obtained on CD4+ T-cell samples using the Illumina HM450 methylation platform as part of the Asthma BRIDGE cohort of 360 CD4+ samples and 690 whole blood samples. Each array assesses methylation at 485,577 CpG sites, including 473,921 autosomal and 11,656 CpG sites from chromosomes X or Y. Quality control (QC) steps were performed by the Channing Division of Network Medicine, and applied norm-exponential background correction on raw data reads for each sample plate. Then the norm-exponential corrected plates were combined and four samples that were outliers were removed. Finally dye-bias correction and further quantile normalization on the above data set was applied.

Thirteen CARE subjects had successful methylation assays. We then removed 43 methylation probes that had missing values for more than half of the remaining subjects. We further excluded 3,091 cross hybridizing probes and 65 SNP probes, resulting in a total of 482,378 methylation marks (135,464 type I CpG sites and 346,914 type II CpG sites). Some subjects had duplicate arrays of CD4+ methylation data; in these cases we analyzed only the array demonstrating the largest inter-quartile range (IRQ). We performed PCA to identify possible batch effects. The first two principal components (PCs) were clearly related to gender and plate effects, which were subsequently adjusted for in the analyses. For each of the 64 metabolites, we tested the association of each CpG site, adjusted for age and the first two methylation principal components, with each metabolite. Type I and type II probes were analyzed separately using statistical routines from the *limma* package available through Bioconductor 24. For each metabolite and for each type of CpG sites, *p*-values for metabolite–CpG association will be adjusted for multiple testing so that false discovery rate (FDR) will be controlled (<0.05).

#### Genome-wide genotyping

We obtained data on genome-wide SNP measurements from the Asthma BRIDGE. Genotyping was originally performed through the SNP Health Association Resource (SHARe) Asthma Resource Project (SHARP), by Affymetrix Inc. (Santa Clara, CA), according to manufacturer's protocol using the Affymetrix Genome-Wide Human SNP Array 6.0. This genotype data are archived on the database of Genotypes and Phenotypes (dbGaP) (http://www.ncbi.nlm.nih.gov/gap/) for all CARE participants. This resulted in 400,741 high-quality SNPs, which were included in our association tests.

We performed linear association tests with all 64 metabolites and all 400,741 SNPs under an additive model using PLINK 26 (v1.07) on the full cohort of 20 CARE subjects. Missing metabolite values were imputed with the mean value for that metabolite, to bias toward the null hypothesis while preserving as much of the cohort as possible. Association tests were modeled as linear regressions with metabolite concentration using age and sex as covariates.

Table S8 shows if a subject had genotype data, metabolite data, expression data, or methylation data. There are nine samples having all four types of data.

### Network methods

#### Overview

**Figure 1:**
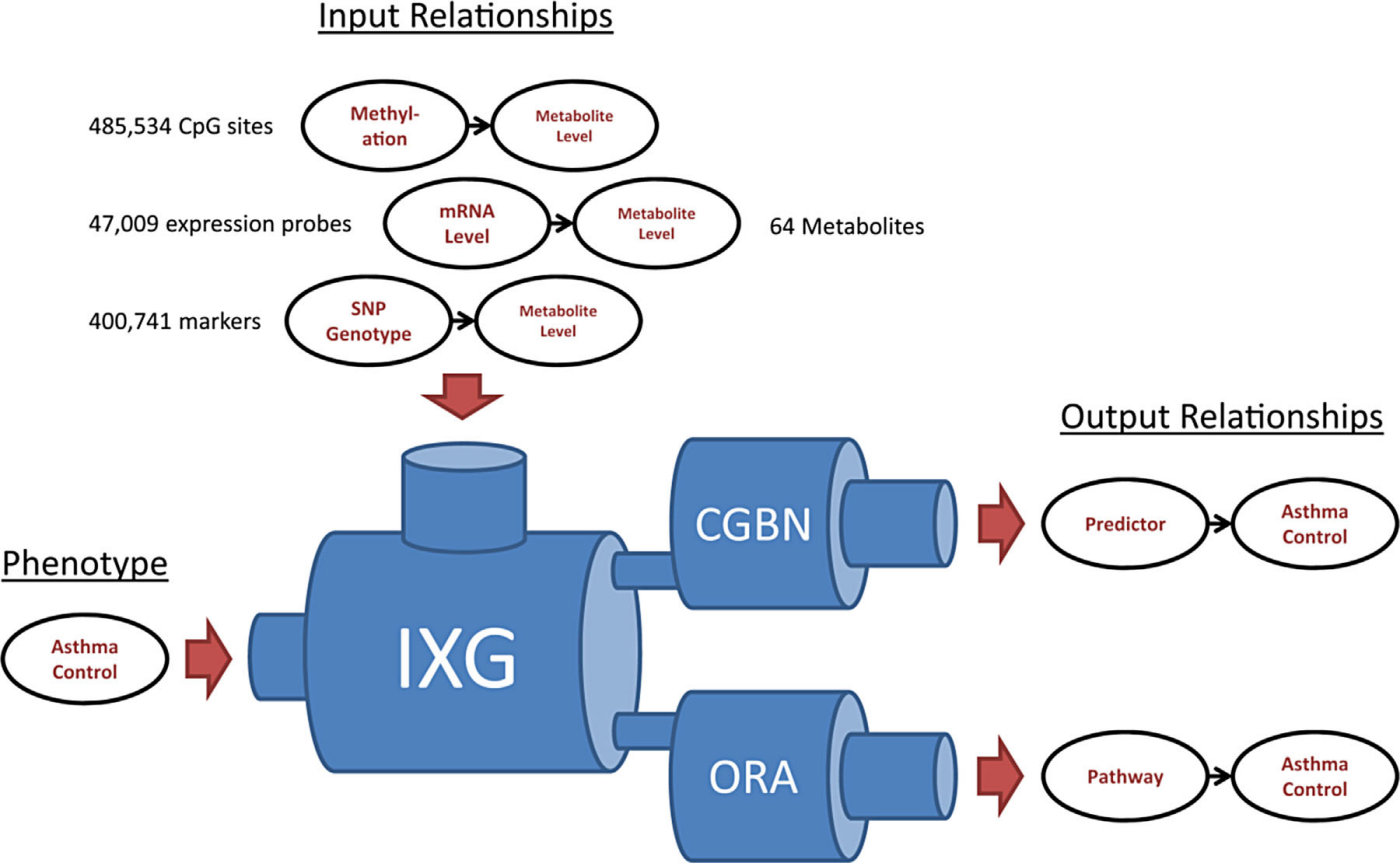
Overview of study design. Directed relationships between top genomic predictors, top metabolites, and asthma control phenotypes were used as input for an integrative genomics pipeline. The two outputs of this pipeline were relationships of pathways and Bayesian predictors, respectively, with asthma control. IXG, integrative genomics; CGBN, conditional Gaussian Bayesian networks; ORA, over-representation analysis.

Figure 1 summarizes our overall integrative genomic approach. We first performed genome-wide analyses using SNPs, mRNA gene expression probes, and CpG methylation sites for all 64 metabolites as phenotypic outcomes. The top association results from each of these analyses, all metabolites, and the asthma control phenotype were used as input into the integrative genomics analyses. Two such analyses were performed. The first analysis was a statistically driven conditional Gaussian Bayesian Network (CGBN) analysis, where variants that contribute to the predictive accuracy of the asthma control phenotype were identified. The second analysis was a knowledgebase-driven overrepresentation analysis (ORA) that identified key pathways that are enriched in individuals with poorer asthma control.

#### Bayesian networks

The expression, SNP, and methylation probes were each ranked according to most significant *p*-value. The top 20 associations to one or more metabolites were selected in combination with the 64 metabolites to be used in a Bayesian network analysis. SNPs in tight linkage disequilibrium (*D′* = 1) were filtered, and missing CpG methylation values were imputed with the mean value, to bias toward no association. Continuous variables were then normalized. This resulted in a dataset of 64 metabolites, 19 SNPs, 19 CpG methylation sites, and 20 mRNA gene expression probes, as well as age and sex, combined with the asthma control phenotype for the full 20 CARE patients.

A conditional Gaussian Bayesian network (CGBN) was learned from this data using the CGBayesNets package in MATLAB version R2013b (MATLAB, The Mathworks Inc., CGBayesNets 27, www.cgbayesnets.com). CGBN is a machine learning technique that makes a network model of the data using the statistical (Bayesian conditional independence) relationships present in the dataset 28. Similar CGBN methodologies have been effective in generating predictive models of intensive care-unit mortality from metabolomic profiling 29. The CGBN was learned using a low uniform Dirichlet prior (and γ prior for continuous nodes) to keep the number of edges low; a technique that has been shown to be effective in prediction in genomic contexts 30 and induces a sparse network with a high complexity penalty 31. We then performed 250 bootstrap realizations of the dataset; a technique that helps correct for small data samples by reducing the effect of outliers within the population. For each bootstrap realization, we learned an exhaustive network using a greedy search that maximized the posterior likelihood of the data given the network. This algorithm starts with an empty network and at each step adds the edge that maximized the posterior likelihood, out of all possible edges. Further details can be found in McGeachie, Chang, and Weiss 27. To obtain a final consensus network from the bootstrap networks, we started with an empty network and added edges sequentially starting with the most commonly occurring in bootstrap samples, and proceeding to less commonly occurring edges, and in each case measured the area under the curve (AUC) of the network so obtained by using fivefold cross validation (CV) on the 20 samples. The network that maximized the CV AUC (AUC = 95.31%) was obtained using an edge threshold of 10.4%, a network that included edges occurring in at least 10.4% of all bootstrap sample networks.

#### Metabolite pathway analysis

For pathway over-representation analysis (ORA) of case-control metabolite data manner, Metabolomics Pathway Analysis (MetPA) (http://metpa.metabolomics.ca/MetPA/faces/Home.jsp) 32 was used. MetPA evaluated a list of the 64 metabolites and their log-normalized concentration data in the 20 samples, by comparing individuals that used rescue albuterol at least once (cases) to those who did not use albuterol (controls). Metabolites were evaluated for pathway enrichment using the “Homo sapiens” library with the default parameters (“Global Test” and “Relative Betweenness Centrality”) specified as the algorithms for pathway enrichment and topological analysis, respectively. The resulting metabolic networks were represented as directed graphs, and centrality measures of a metabolite within a given network were then applied to estimate the relative importance of that metabolite in the network. For topographical analysis, the “pathway impact” score for a given metabolite was then calculated as the sum of the importance measures of that metabolite normalized by the sum of importance measures for all nodes within each pathway. Univariate tests were then performed to evaluate the distribution of individual metabolite concentrations in individuals with good and poor asthma control.

#### Integrative pathway analysis

To integrate metabolomic and gene expression data simultaneously, ORA and Wilcoxon pathway enrichment analysis (WEA) were performed using Integrated Molecular Pathway Level Analysis (IMPaLA) (http://impala.molgen.mpg.de/) 33. We selected the top 20 gene probes based on their association *p*-values with any of the given metabolites. A list of the 64 metabolite IDs, gene IDs, and fold differences in metabolite concentrations and gene expression values for cases and controls were uploaded into the IMPaLA webserver, specifying the metabolites and genes present across all pathways in the database as the background list for comparison. For ORA, the hypergeometric distribution with BH-adjustment of *p*-values was applied to determine the significance of pathways that overlap with the metabolite and gene query lists. For WEA, the Wilcoxon test was used. Joint *p*-values for genes and metabolites were calculated for each pathway using Fisher's method. For both approaches, only pathways with one or more overlapping gene and metabolite pair were evaluated. Pathways with unadjusted *p-*values <0.05 were considered significant. *p*-Values were corrected for multiple testing using the FDR (threshold of 0.05).

Genes were also mapped to pathways independently of phenotype information, using WebGestalt (http://bioinfo.vanderbilt.edu/webgestalt/) webserver tools 34, specifying the human genome as a reference list, and using the hypergeometric distribution with BH-adjustment to evaluate the significance of pathways. To investigate pharmacogenetic pathways, pathway analysis was performed using WebGestalt to query the drug-associated genes annotated in the database, PharmGKB 35. For all results, pathways with unadjusted *p-*values <0.05 were considered significant. Graphs of gene networks were generated using GeneMania 36.

#### In silico validation using the connectivity map

The Connectivity Map (www.broad.mit.edu/cmap) (CMAP) is a publicly available, searchable database of gene expression profiles collected from treatment of human cell lines with small molecules 37. A gene-expression signature for “salbutamol” (a.k.a. albuterol) was generated using data from two independent experiments conducted in PC3 cells, specifying probe expression threshold values of 0.67 to −0.67 (corresponding to twofold changes in gene expression) for up- and down-regulated probe sets. This signature was used to query CMAP for compounds with correlated effects on gene expression, emphasizing the lipid metabolites evaluated in this study. A gene signature for “dinoprostone” (a.k.a. PGE2) was similarly generated, and genes within the signature were then evaluated for metabolite pathway enrichment using WebGestalt.

## Results

### Description of the study cohort

Descriptive and clinical characteristics of the cohort are shown in Table1, stratified by SABA use. A cutoff of zero versus one or more uses of SABA to identify cases and controls was a natural point of dichotomy for a pharmacological outcome. Subjects in the two categories did not differ significantly, apart from SABA use.

**Table 1:** Demographic and descriptive characteristics of study subjects

	Frequency of albuterol use in last 7 days		
	None (*n *= 12)	≥1 (*n *= 8)	*p-*value
Age (mean, range)	13.3	14.9	0.77
Age at asthma symptom onset (mean, range)	2.5	3.8	0.79
Gender (% female)	25	50	0.25
Race (% self-reported European ancestry)	100	100	1.00
Allergic rhinitis (%)	50	88	0.09
Eczema (%)	42	63	0.36
Food allergy (%)	33	25	0.69
Use of inhaled corticosteroids in last 7 days (%)	42	50	0.71

Baseline characteristics of study subjects are shown, stratified by exacerbation phenotype. *p-*Values are computed using *χ*^2^ test of Fisher exact test, as appropriate.

### Metabolite profiling

**Figure 2:**
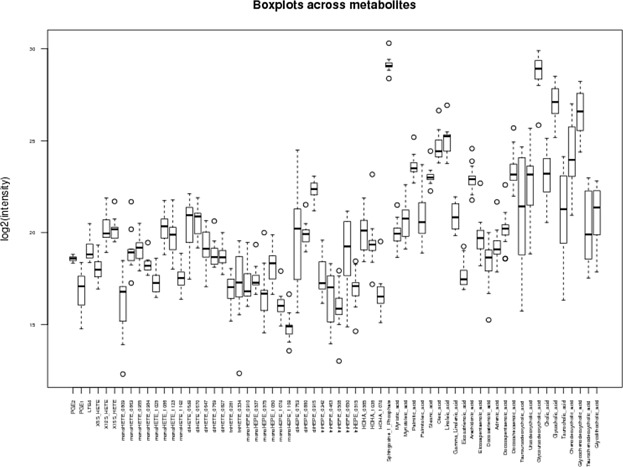
Concentrations (log_2_) across 64 metabolites for 16 CARE subjects. The distribution of each metabolite across the study cohort is presented as a boxplot.

The characteristics of all 64 lipid metabolites are presented in Table S1. The distributions of metabolite concentrations are shown in Figure 2. While metabolite concentrations varied greatly across the cohort, the metabolite concentrations were not significantly different between subjects, with or without SABA use, after adjusting for multiple testing, although decreases in monoHETE_0863 were nominally associated with well-controlled asthma (*p* = 0.015). To further investigate the correlation structure between various metabolites and the case/control status of our cohort, we conducted unsupervised two-dimensional hierarchical clustering of the metabolite concentrations by CARE participant (Figure S1). While none of the subjects could be differentiated into groups by SABA use based on correlated metabolite clusters, some of the individual metabolites were correlated with each other. The correlated metabolites included tauroursodeoxycholic acid (a.k.a. ursodeoxycholyltaurine) and taurocholic acid (*r*^2^ =0.95), which are both bile acids. In addition, there was a cluster of four correlated metabolites: stearic acid, docosapentaenoic acid, adrenic acid (a.k.a. all-cis-7,10,13,16-docosatetraenoic acid), and palmitic acid (all *r*^2^ > 0.9); stearic acid and palmitic acid are common saturated fatty acids produced from triglyceride and carbohydrate metabolism, while docosapentaenoic acid and adrenic acid are eicosanoids derived from α-linoleic acid and arachidonic acid, respectively. Docosahexaenoic acid and eicosatrienoic acid (*r*^2^ = 0.93), both eicosanoids, were also tightly correlated. No other metabolite correlations were |*r*^2^| > 0.9. Finally, a group of five bile acids (taurochenodeoxycholic acid, tauroursodeoxycholic acid, taurocholic acid, glycocholic acid, and glycoursodeoxycholic acid) were anti-correlated with the majority of other metabolites (Figure S1; five right-most columns). These data show that while specific metabolite concentrations could be correlated with one another across samples, the case and control groups could not be differentiated from each other based solely on the correlation of metabolite concentrations.

### Pathway analysis of metabolites

**Figure 3:**
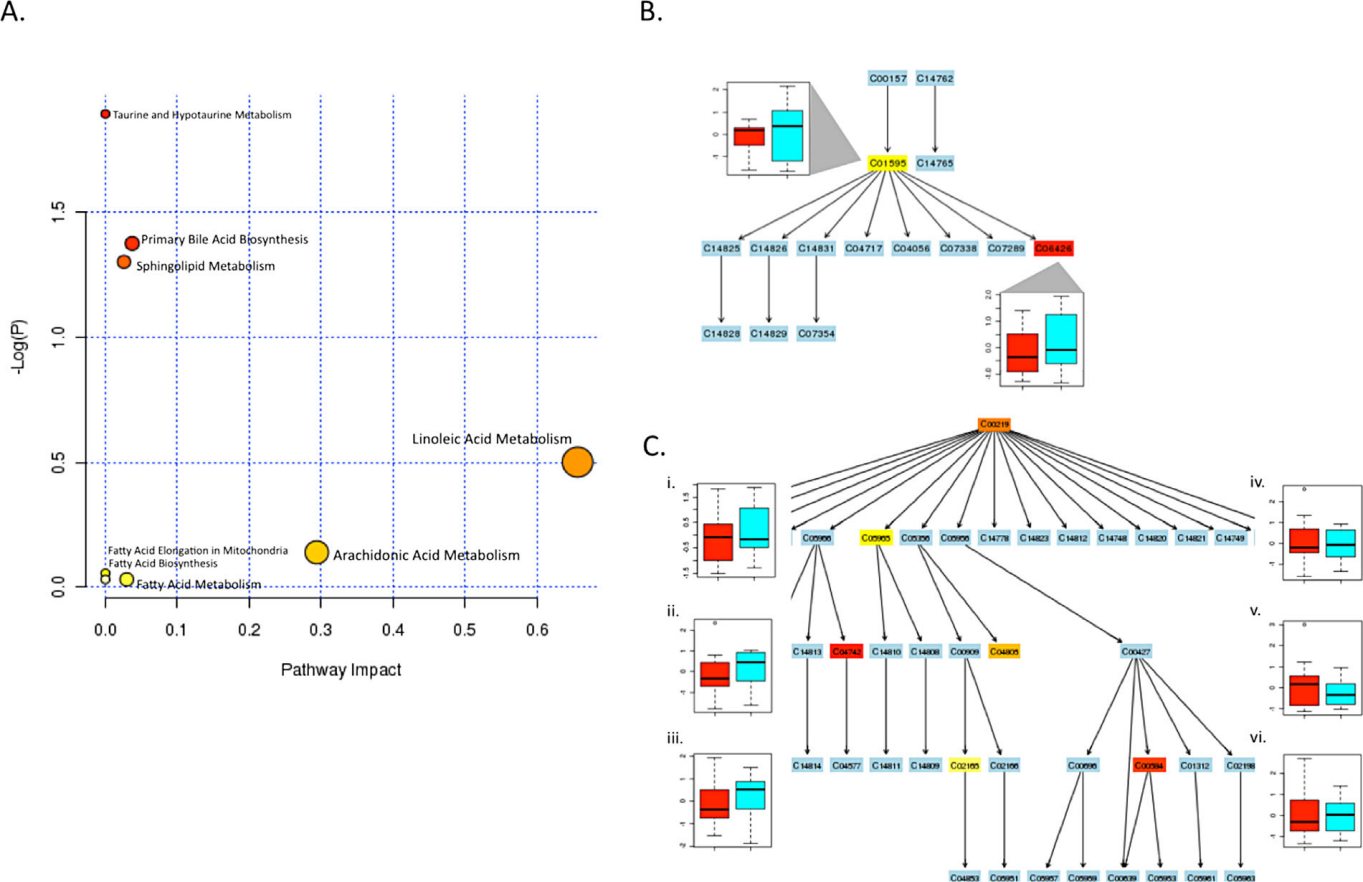
Metabolomics pathway analysis. (A) Plot shows metabolome view of pathway enrichment analysis results in the asthma cohort. In side panels, metabolic pathways are shown for (B) “Linoleic Acid Metabolism” and (C) “Arachidonic Acid Metabolism”. In B and C, labels within small boxes correspond to KEGG identifiers for metabolites. Box color gradient indicates increasing significance values for a given metabolite within the pathway from least (blue) to the most significant (red). Boxplot figures in B and C represent the median ± IQR for log-normalized concentrations for the indicated metabolite, in the cases (teal boxplots) versus controls (red boxplots). In B, corresponding boxplots for linoleic acid (C01595) and γ-linoleic acid (C06426) concentrations are shown. The numerals in C correspond to (1) arachidonic acid (C00219), (2) 5-HETE (C04805), (3) PGE2 (C00584), (4) 12(S)-HPETE (C05955), (5) 15(S)-HETE (C04742), (6) LTB4 (C02165).

None of the pathways were significantly associated with asthma control (Table2). As a result, the pathway impact scores were used instead of *p*-values to rank pathways in order of priority. Five pathways had impact scores above 0. The “Arachidonic Acid Metabolism” and “Linoleic Acid Metabolism” pathways possessed the greatest number of overlapping metabolites and the highest pathway impact scores (Fig. 3A). Within the “Linoleic Acid Metabolism” node, two overlapping query metabolites, linoleic acid (KEGG: C01595) and γ-linoleic acid (KEGG: C06426), showed higher concentrations in the cases, although these differences were not significant (Fig. 3B). Similarly, for the arachidonic acid pathway, six overlapping metabolites (arachidonic acid (KEGG: C00219), 5-HETE (KEGG: C04805), PGE2 (KEGG: C00584), 12(S)-HPETE (KEGG: C05955), 15(S)-HETE (KEGG: C04742), and LTB4 (KEGG: C02165)) demonstrated differential concentrations in cases and controls, but these differences were not significant (Fig. 3C).

**Table 2:** Metabolomic pathways

Pathway name	Total no. of metabolites	No. of overlapping metabolites	Unadjusted *p-*value	FDR-adjusted *p-*value	Pathway impact score
Linoleic acid metabolism	15	2	0.61	0.97	0.66
Arachidonic acid metabolism	62	6	0.87	0.97	0.29
Primary bile acid biosynthesis	47	6	0.25	0.72	0.04
Fatty acid metabolism	50	1	0.97	0.97	0.03
Sphingolipid metabolism	25	1	0.27	0.72	0.03
Taurine and hypotaurine metabolism	20	1	0.15	0.72	0.00
Fatty acid biosynthesis	49	5	0.94	0.97	0.00
Fatty acid elongation in mitochondria	27	1	0.97	0.97	0.00

### Association of metabolites with “omics” data

#### SNP–metabolite associations

Although our low sample size lead to underpowered interrogation of SNP–metabolite associations, four had FDR < 0.05. Further, of 64 × 400,741 SNP-metabolite association tests, 31 associations were present with an FDR < 0.20 (shown in Table S2). Since the low power may miss some important associations, we chose a more lenient FDR 0.20 cutoff, which allowed us to prioritize a selection of top associations for further analysis. Of these 31 associations, two are in or near genes that are crucial for, or directly participate in, the metabolism of the metabolite(s) (*ATP10* and *HAO2*, respectively) (Table S2). As members of the type-IV subfamily of p-type ATPases are aminophospholipid translocases 38, *ATP10* may participate in transport of arachidonic pathway precursors (phospholipids) that are metabolized to adrenic acid 39. Similarly, co-association of *HAO2* and docosahexaenoic acid is appropriate because the gene is required for metabolism of fatty acids within this pathway 40. Direct relationships for the remaining loci with their associated metabolites were less evident.

#### Gene expression–metabolite associations

We next investigated the relationship between mRNA expression and metabolite concentrations. Of the 64 × 47,009 mRNA–metabolite associations, the 20 strongest associations, ranked by FDR, are shown in Table S3. The eicosanoid docosatrieneoic acid was the most frequently observed metabolite among the gene–metabolite pairs, and was associated with expression of eight genes. The bile acid metabolites ursodeoxycholyltaurine and ursodeoxycholylglycine were linked to expression of four genes, and docosapentaneoic acid and glycocholic acid were connected to single genes (Table S3). An uncharacterized metabolite, HDHA_1074, was linked to five genes (Table S3). While there were no statistically significant associations (FDR < 0.05), in order to prioritize a selection of top hits for further analysis, we included the top 20 mRNA expression probes in the Bayesian network methodology. In this case, without a clear FDR cutoff presenting itself, we chose the top 20 to be similar to the number of top selections identified from other integrative “omics” analysis.

#### CpG methylation–metabolite associations

We computed associations of methylation at individual CpG sites to each of the 64 metabolites, adjusting for age and the first two PCs. Type I CpG sites and type II CpG sites were analyzed separately. For type I CpG sites, the numbers of significant tests (FDR-adjusted *p*-value <0.05) for the 64 metabolites were ranged from 0 to 1,463. Fifty-nine metabolites had at least one significant association. The metabolite *Taurochenodeoxycholic_acid* had the maximum number of significant metabolite-type-I-CpG association tests. For type II CpG sites, the numbers of significant tests for the 64 metabolites were ranged from 0 to 3,759. Fifty-nine metabolites had at least one significant association. The metabolite *Taurochenodeoxycholic_acid* had the maximum number of significant metabolite-type-II-CpG association tests. We kept the top 50 metabolite-type-I-CpG and metabolite-type-II-CpG association tests, each of which contained nine unique metabolites. There were 12 unique metabolites in the two sets of nine metabolites. For each of the 12 metabolites, we selected the top two associated CpG sites for further analysis and inclusion in our Bayesian network methodology. This resulted in 19 total methylation markers (Table S4). The markers were annotated to 19 genes, which included *GLCCI1* (which was also associated with taurocholic acid), an important candidate gene that modulates responses to steroid treatment in asthmatics 41.

### Integrative pathway analysis of metabolite–gene associations

**Figure 4:**
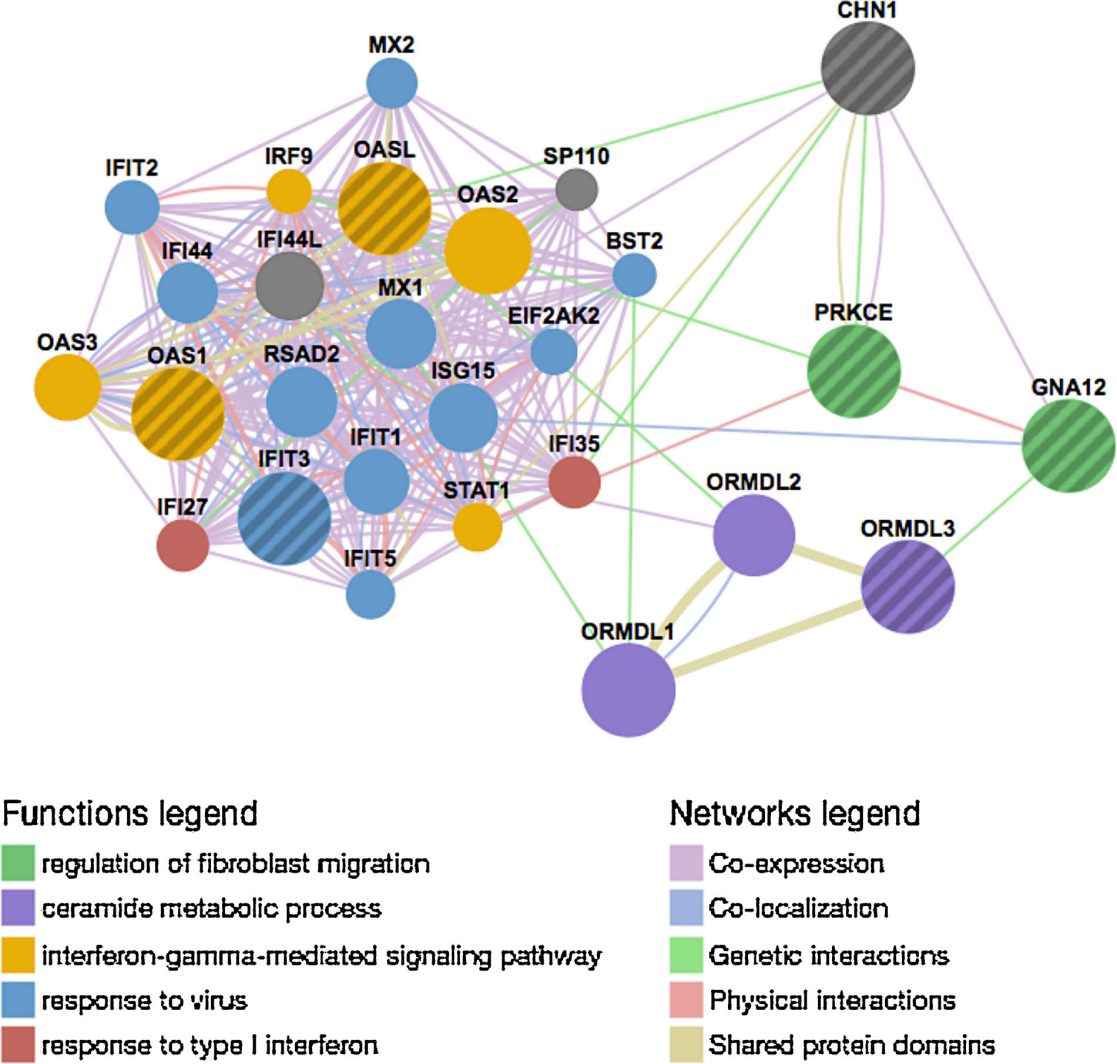
Network of molecular interactions for integrative ORA pathway genes. A network was generated for *CHN1*, *PRKCE*, *GNA12*, *OASL*, *OAS1*, *ORMDL3*, and *IFIT3*. Nodes (circles) represent genes, and lines between nodes (edges) represent relationships (co-expression, co-localization, shared protein domains, physical and genetic interactions) between nodes. Node color indicates pathway annotation, and the strength of the evidence in support of the indicated interaction is shown by edge thickness.

Docosatrienoic acid had the greatest number of associations with individual genes, and was also among the top-ranked associations (Table S3), providing additional evidence that eicosanoid pathways for linoleic acid and arachidonic acid (Table2 and Fig. 4) are important network hubs. We investigated the biological relevance of the 20 mRNA–metabolite relationships (shown in Table S3) using IMPaLA to integrate gene expression and metabolite data.

Twenty-seven metabolites, and 11 of the genes shown in Table S3, mapped to 17 distinct molecular pathways (the top 10 pathways are listed in Table3). The most frequently observed metabolite was arachidonic acid, which appeared in 12 of the 17 pathways, singly or among related metabolites (Table3). In addition, PGE2 and sphingosine-1-phosphate (S1P) were the second and third most highly represented metabolites among the pathways (Table3). Thus, the ORA results were driven by the presence of three metabolites that either directly represent (or are known to modulate) the eicosanoids, namely arachidonic acid, PGE2 and S1P.

**Table 3:** Top 10 pathways for integrative ORA of genes and metabolites

Pathway name	No. of overlapping genes	No. of overlapping metabolites	Gene *p-*value	Metabolite *p-*value	Joint *p-*value	Adjusted joint *p-*value	Gene symbol	Metabolites
Signaling by GPCR	2	17	0.31	1.65E−20	2.42E−19	4.00E−17	*GNA12*, *PRKCE*	PGE2, arachidonic acid, cholic acid, palmitic acid, palmitoleic acid, all-cis-7,10,13,16-docosatetraenoic acid, stearic acid, gamma-linolenic acid, myristic acid, sphingosine 1-phosphate, docosapentaenoic acid, leukotriene B4, chenodeoxycholic acid, eicosatrienoic acid, oleic acid, eicosapentaenoic acid, docosahexaenoic acid
Signal transduction	3	18	0.32	1.02E−19	1.47E−18	9.72E−17	*GNA12, PRKCE, CHN1*	Eicosatrienoic acid, arachidonic acid, cholic acid, palmitic acid, chenodeoxycholic acid, all-cis-7,10,13,16-docosatetraenoic acid, stearic acid, gamma-linolenic acid, myristic acid, sphingosine 1-phosphate, docosapentaenoic acid, leukotriene B4, palmitoleic acid, linoleic acid, PGE2, oleic acid, eicosapentaenoic acid, docosahexaenoic acid
Inflammatory mediator regulation of TRP channels—homo sapiens (human)	1	6	0.10	7.06E−08	1.37E−07	1.64E−06	*PRKCE*	12(S)-HPETE, 15(S)-HETE, leukotriene B4, arachidonic acid, 5-HETE, PGE2
GPCR downstream signaling	2	8	0.26	9.16E−08	4.46E−07	5.07E−06	*GNA12, PRKCE*	Cholic acid, palmitic acid, leukotriene B4, sphingosine-1-phosphate, arachidonic acid, oleic acid, PGE2
G-α (q) signaling events	1	5	0.18	5.00E−06	1.36E−05	0.000118	*PRKCE*	Palmitic acid, leukotriene B4, oleic acid, arachidonic acid, PGE2
Gastrin-CREB signaling pathway via PKC and MAPK	1	5	0.20	5.00E−06	1.49E−05	0.000126	*PRKCE*	Palmitic acid, leukotriene B4, oleic acid, arachidonic acid, PGE2
Fc-γ R-mediated phagocytosis—homo sapiens (human)	1	2	0.091	0.001	0.001	0.005	*PRKCE*	Sphingosine-1-phosphate, arachidonic acid
S1P5 pathway	1	1	0.008	0.019	0.002	0.007	*GNA12*	Sphingosine 1-phosphate
S1P4 pathway	1	1	0.015	0.019	0.003	0.011	*GNA12*	Sphingosine 1-phosphate
S1P2 pathway	1	1	0.027	0.026	0.006	0.022	*GNA12*	Sphingosine 1-phosphate

The largest and best-ranked pathway was “signal transduction” (Table3), which contained 18 metabolites including the docosatrieneoic acid precursor, docosahexaenoic acid, as well as other linoleic acid pathway metabolites. In addition, several pathways contained genes whose expression was associated with this metabolite, including *CHN1*, *IFIT3*, *OASL*, and *OAS1*, which were present in multiple pathways for immune response, IFN and cytokine-related signaling (Tables S3 and S4). The pathways also included the genes *GNA12* and *PRKCE*, of which *PRKCE* and *GNA12* were well-represented in the top pathways (Table3). Finally, through querying PharmGKB^35^, we confirmed that *OASL* and *OAS1* also represent pharmacogenetic loci for interferon-based drugs (data not shown).

Integrative ORA implicated multiple pathways related to interferon signaling and sphingolipid signaling that included *PRKCE* and/or *GNA12*. As multiple genes were annotated to the same or redundant pathways, they are likely to have important, and possibly complementary, roles in these immune response pathways. Using *CHN1*, *PRKCE*, *GNA12*, *OASL*, *OAS1*, and *IFIT3* as query genes, we generated a network (GeneMania 36) of molecular interactions. Interestingly, this gene network also interacted with an important asthma susceptibility gene, *ORMDL3* 7,42–44, and two of its homologs, *ORMDL1* and *ORMDL2*. Genetic interactions exist between *ORMDL3* and *GNA12*, and between *OASL* and *ORMDL2*, suggesting that these genes interact within a cellular network (Fig. 4, green lines) 45.

### Bayesian network results

In parallel with the ORA-based approach, we implemented a more statistically driven approach to identify predictors of asthma control. To this end, we combined the 64 metabolites with top association results from the SNP, mRNA expression, and CpG methylation analysis into a single integrative “omics” dataset, and generated a conditional Gaussian Bayesian network (CGBN) predictive of asthma control among the 12 cases and 8 controls in CARE (Fig. 1).

**Figure 5:**
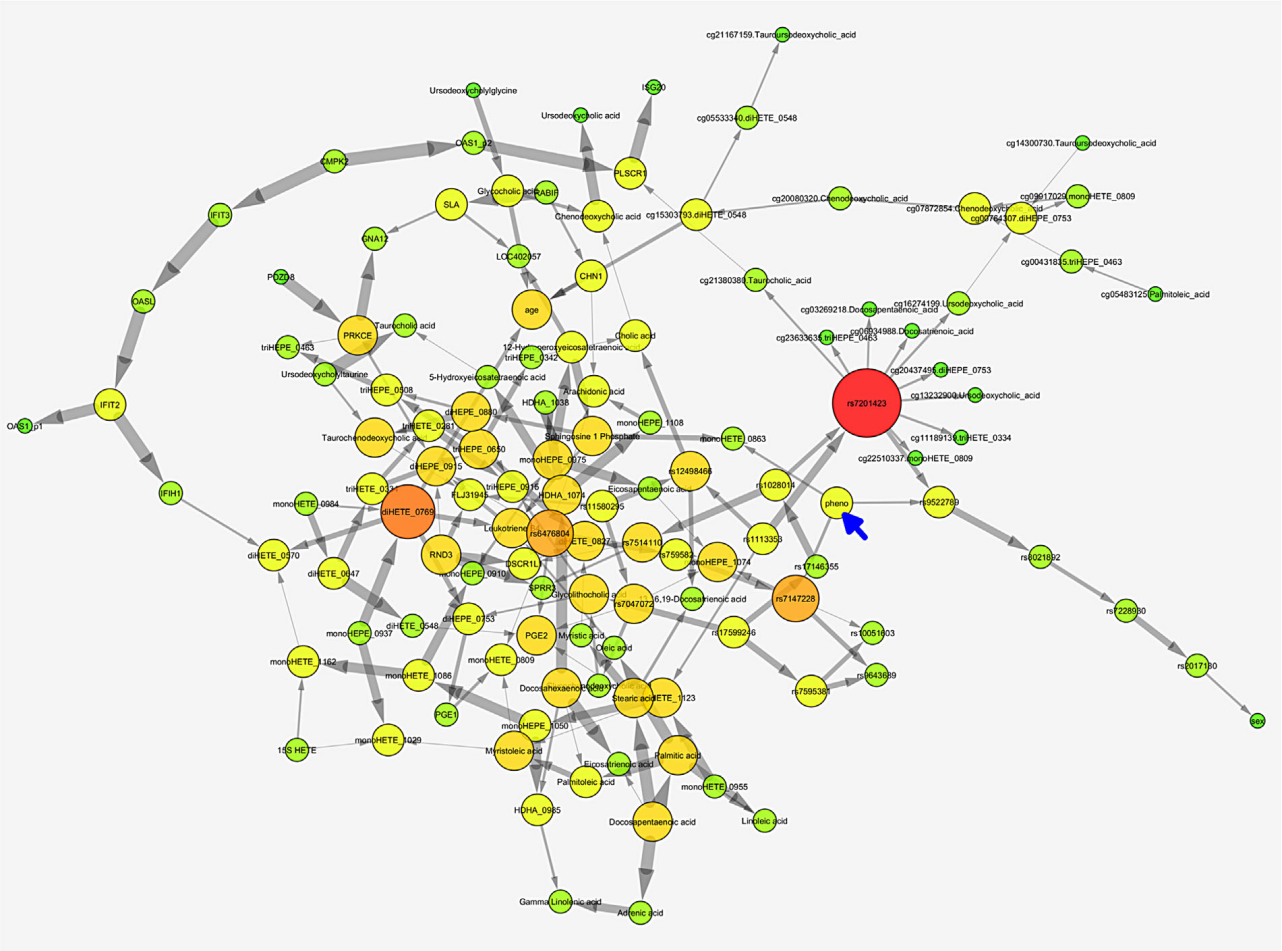
Consensus Bayesian network. Nodes represent gene expression probe levels, CpG site methylation percents, SNP minor allele distributions, and metabolite levels. The phenotype, asthma control, is marked with a blue arrow. Nodes with more connections are bigger and redder; gray arrows between nodes indicate the Bayesian conditional independence of the child node given the parent nodes of the remaining nodes. Thicker arrows represent stronger statistical dependence.

After building a CGBN using bootstrapping and cross-validation (see Methods), we obtained a consensus network (Fig. 5), using a low-edge inclusion threshold from the bootstrap networks (10.4%). This low threshold is indicative of a variety of different networks generated in bootstrapping and which is generally a consequence of the low sample-size. The consensus network was successful at predicting asthma control in the 20 sample cohort with a CV AUC of 95.31%. Prediction by a CGBN of a phenotype (blue arrow, Fig. 5) requires only the nodes within the Markov Neighborhood of the phenotype (the node's children, and other parents of those children). In this case, the predictive ability of the CGBN is based on two metabolites, Sphingosine 1 Phosphate (S1P) and monoHETE_0863; and four SNPs, rs9522789 (far from genes), rs7147228 (intronic to *CDKL1*), rs7201423 (in *LOC101927131*), rs759582 (near *CMAS*). SNP rs759582 is in tight LD (*r*^2^ = 0.95) with a SNP, rs56069081, in a DNAseI hypersensitivity site in a sample of lung tissue tumor (HaploReg v3, ref). SNP rs9522789 is in tight LD with rs1578536 (*r*^2^ = 0.87), also in a DNAseI hypersensitivity site, and one marked with enhancer histones in vascular endothelial cells (HaploReg). It is possible these SNPs have regulatory effects, although they are intergenic.

The CGBN network was compared with results from ORA to determine whether the biologically inferred and statistically based methods could validate one another. Of the CBGN predictors, multiple pathways that included S1P as a major metabolite were identified from the metabolomic and integrative ORAs. For example, from the metabolomics ORA of 31 metabolites, “sphingolipid metabolism” had been identified (Fig. 3A). Furthermore, out of 17 pathways identified in the integrative ORA, nine pathways included S1P (Table3). These pathways also included the genes *GNA12* and *PRKCE*, the latter of which also constitutes a network hub within both CGBN (Fig. 5) and a molecular interaction network (Fig. 4) that is also predicted to interact with *GNA12*.

### The S1P pathway is modulated in response to albuterol: in silico validation using CMAP

To provide insight into potential cellular mechanisms for asthma control, we searched CMAP 37 to identify lipid metabolites with gene expression signatures that were significantly correlated with those resulting from albuterol (“salbutamol”) treatment, indicating a common mechanism of action.

The CMAP query identified 96 small molecules that were significantly correlated (*p* < 0.05) with the gene expression signature for “salbutamol,” a.k.a. albuterol. Among these were PGE1 (“alprostadil”) and PGE2 (“dinoprostol”), which were significantly positively correlated with genes responsive to albuterol (*p* = 2–4 × 10^−05^) and were also ranked among the top 10 correlated compounds (Table S6). As our pathway-based and CGBN analyses both implicated a role for S1P, which stimulates pro-inflammatory function via boosting production of PGE2 (the major inflammatory mediator prostaglandin), we investigated expression of genes within the albuterol gene signature that were also upregulated (*n* = 126) or down-regulated (*n* = 169) in response to treatment with 10 μM PGE2. The gene encoding prostaglandin E synthase (*PTGES*) was the most highly upregulated gene, while *ADRB2*, the molecular target of albuterol, was down-regulated; as PGE2 negatively regulates *ADRB2*, this finding was consistent with previous reports of these interactions (data not shown). Interestingly, 2 of the 20 candidate genes, *ISG20* and *OASL*, were also present and highly upregulated (>3.5-fold) in this data set (data not shown). Importantly, the S1P pathway (Pathway Commons Pathway “Sphingosine-1-phosphate (S1P) pathway”) was significantly enriched in the PGE2 expression signature (adjusted *p* = 2.2 × 10^−21^). Thirteen genes annotated to the S1P pathway were up-regulated (>1.5-fold), while eight genes were down-regulated (Table S7).

## Discussion

Our study has four key findings. First, our integrative “omics” analysis implicates two pathways—arachidonic acid metabolism and linoleic acid metabolism—in asthma control. Second, a Bayesian network of the integrated dataset predicts uncontrolled asthma with high CV accuracy, supporting the validity of our integrative approach to identify important metabolite and gene interactions. Third, altered sphingolipid metabolism appears to be an underlying feature of uncontrolled asthma and cellular response to albuterol. Finally, our results suggest that albuterol and PGE2 share common cellular pathways, based on correlated gene signature expression patterns that include genes involved in S1P metabolism and/or activity.

The metabolic pathways for the eicosanoids arachidonic acid and linoleic acid had the highest impact scores. These two pathways also scored highest for metabolite–mRNA associations, with the eicosanoid docosatrieneoic acid represented among the most frequently observed metabolites. Although these results were not significant, the lack of statistical significance was likely due to our small sample size. Despite this, these findings still have relevance for asthma, as arachidonic acid is the precursor for eicosanoid production, including leukotrienes and prostaglandins, and both 15(S)-HETE and PGE_2_ are differentially produced during acute exacerbations 46. In addition, linoleic acid metabolites also induce airway hyper-responsiveness 47 and variable linoleic acid exposure influences asthma symptoms 48. Integrative ORA identified multiple metabolites with important relationships to genes involved in smooth muscle contraction, which is an important mechanism of bronchial hyper-responsiveness and/or airway constriction, and inflammation. Arachidonic acid can alter inflammatory gene expression through *PRKCE* 49,50 and G-protein coupled receptor and GTP-ase activity, potentially via *GNA12* and *CHN1*.

The Bayesian Network based on four SNPs and two metabolites predicts asthma control with high CV accuracy, supporting our hypothesis that inclusion of multiple omics would lead to greater accuracy and while also revealing important relationships among the metabolites and top SNPs, genes, and epigenetic marks. Of course, considering the low sample size in the current work, and the large network assembled, we cannot rule out overfitting as a cause of predictive accuracy, although the bootstrapping and cross-validation methods are designed, along with the Bayesian priors, to guard against this. We feel that these findings provide evidence to support the ability of our methods to identify important metabolite and gene interactions, and further implicate sphingolipid signaling as an important metabolomic signature for asthma control. Furthermore, of these predictors, S1P, was enriched in the results from integrative and metabolic ORAs, demonstrating that both the biologically inferred and statistically based approaches we applied were complementary, and could serve to validate one another.

Evidence from both methods implicated sphingolipid metabolism; in particular, the BN subnetwork of *GNA12*, *PRKCE* and S1P, may represent a potential mechanism for asthma control. *GNA12* encodes guanine nucleotide binding protein (G-protein) α 12, which couples to the lung and lymphoid-tissue specific S1P receptor, *S1P*(*4*), in response to S1P binding 51. GNA12 also physically interacts with, and is phosphorylated by, PRKCE 52. In turn, these genes interact with a variety of signaling molecules and binding partners, including genes identified in our analysis such as *CHN1*, and the interferon-responsive *OAS* and *IFIT3* genes, in order to effect cellular responses. Our finding that altered sphingolipid metabolism is important in uncontrolled asthma is supported by several reports describing the role of sphingolipids in airway inflammation and asthma, and as therapeutic targets 53,54. Furthermore, we explored the potential interaction of the network genes with *ORMDL3*, a negative regulator of sphingolipid biosynthesis with upregulated expression in asthmatics 7. ORMDL3 also contributes to airway hyperreactivity through altered sphingolipid biosynthesis 55. This study implicates a role for genes that participate in eicosanoid and sphingolipid signaling, chiefly *GNA12* and *PRKCE*, that also interact with *ORMDL3* and interferon-responsive *OAS* and *IFI* gene family members, in a molecular interaction network.

As a first step toward functional validation, we investigated these potential interactions in silico using CMAP, a publicly available reference database of gene expression profiles from human cell lines treated with small molecules that can be mined for shared biological connections. In this analysis, albuterol gene expression signatures serve as a cellular proxy for asthma control, and molecules that demonstrate significantly shared expression signatures with albuterol are anticipated to share mechanisms of action. We determined that the gene expression signatures for albuterol were highly correlated with PGE2, and that *ISG20* and *OASL* were both upregulated in response to treatment with both compounds. Furthermore, 21 additional genes within the S1P metabolic pathway were differentially expressed.

To our knowledge, few metabolomic studies have been performed for asthma; most have focused on asthma diagnosis while two studied asthma severity 14–21. In general, these studies had similarly good predictive accuracy (>80%) in differentiating asthmatics with good and poor control in our study 14–17. None of the prior studies implicated altered sphingolipid metabolism. Saude et al. identified five metabolites in the tricarboxylic acid (TCA) cycle with a higher abundance in subjects who experienced exacerbations compared to subjects who did not experience exacerbations 17,18. This is consistent with the histamine release that occurs during mast cell activation in asthma exacerbations 18. A genomic analysis was also conducted using serum from asthma patients experiencing serious asthma exacerbations to identify the transcriptional variation during the process of an exacerbation, which also identified immune-related genes associated with uncontrolled asthma 56. This finding and others are promising, identifying metabolites related to TCA cycle metabolism, hypoxic stress, immune reaction and inflammation, all of which are biologically plausible metabolites for asthma 14,18.

Notably, our investigation represents the first integrative metabolomics study of asthma, in addition to the first asthma pharmacometabolomics study conducted to date. Our approach uses metabolites as intermediate measures to connect each of the other “omics” data sets. A limited set of the remaining “omics” was then tested relative to the clinical outcome. By using either the ORA-based or CGBN integrated approach, our analysis shows that novel predictors of asthma control can be reliably identified. Despite the strengths of our study, a few potential weaknesses deserve mention. The primary weakness of this study is the limited sample size. Despite this, both the statistical and bioinformatics pathway analyses have consistent results. In addition, there were no clinical, baseline, or descriptive differences between our cases and controls, suggesting that the two groups are well matched. The small sample size and lack of power likely contributed to our inability to identify statistically significant associations in some of our single-genomic modalities, such as the mRNA expression and methylation analysis. Investigations using larger samples sizes in the future are warranted. In addition, our study used blood plasma samples for the omic investigation, and it may be of future interest to assess these findings in a respiratory tissue such as lung epithelium or airway smooth muscle, although previous studies have shown that asthma exacerbation is detectable from mRNA expression in blood 56. Finally, the phenotype assessed was based on self-reported albuterol use, which may be a source of bias and represents a limitation of the analysis.

In conclusion, altered sphingolipid metabolism represents an underlying feature of both asthma control and cellular response to albuterol. Lipid mediators play an important role in airway inflammation and asthma, and sphingolipid metabolites serve as novel molecular candidates for future functional validation studies.

## Supporting Information

Figure S1 . Two-dimensional hierarchical clustering of metabolites.

Table S1 . Characteristics of known lipid metabolites identified through metabolomic profiling. **Table S2**. List of top SNP-metabolite associations, for each possible metabolite. **Table S3**. Top 20 mRNA-metabolite associations. **Table S4**. Top metabolite–methylation associations. **Table S5**. Candidate gene pathways. **Table S6**. Connectivity map query. **Table S7**. S1P pathway genes enriched in PGE2 gene expression signature. **Table S8**. Chart showing if a subject had genotype data, metabolite data, expression data, or methylation data.
