# Supplementary figures and images for: The metabolomics of asthma control: a promising link between genetics and disease
Source: Immun Inflamm Dis. 2015 May 7;3(3):224–38. doi: 10.1002/iid3.61 (PMC4578522; doi:10.1002/iid3.61)

**Figure S1.**

**
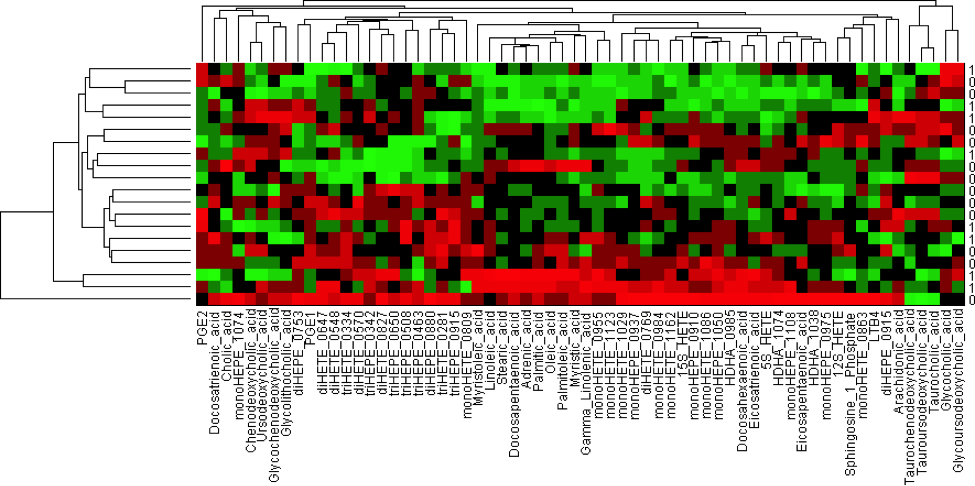
**

Supplement: Figure S1 — . Two-dimensional hierarchical clustering of metabolites. [file iid30003-0224-sd1.docx]
