# Supplementary material for: The metabolomics of asthma control: a promising link between genetics and disease
Source: Immun Inflamm Dis. 2015 May 7;3(3):224–38. doi: 10.1002/iid3.61 (PMC4578522; doi:10.1002/iid3.61)
Supplement: Table S1 — . Characteristics of known lipid metabolites identified through metabolomic profiling. Table S2. List of top SNP-metabolite associations, for each possible metabolite. Table S3. Top 20 mRNA-metabolite associations. Table S4. Top metabolite–methylation associations. Table S5. Candidate gene pathways. Table S6. Connectivity map query. Table S7. S1P pathway genes enriched in PGE2 gene expression signature. Table S8. Chart showing if a subject had genotype data, metabolite data, expression data, or methylation data. [file iid30003-0224-sd2.docx]

**Table S1. Characteristics of Known Lipid Metabolites Identified Through Metabolomic Profiling.**

| **Metabolite Name** | **Type** | **Chemical Class** | **Precursor** |
| --- | --- | --- | --- |
| 12-Hydroperoxyeicosatetraenoic acid | Unsaturated fatty acid | Eicosanoid | Arachidonic acid |
| 13,16,19-Docosatrienoic acid | long-chain omega-3 fatty acid | Docosanoid | docosahexaneoic acid |
| 15-S-Hydroxyeicosatetraenoic acid | Unsaturated fatty acid | Eicosanoid | Arachidonic acid |
| 5-Hydroxyeicosatetraenoic acid | Unsaturated fatty acid | Eicosanoid | Arachidonic acid |
| all-cis-7,10,13,16-docosatetraenoic acid | omega-6-fatty acid | Eicosanoid | Arachidonic acid |
| Arachidonic acid | omega-6-fatty acid | Eicosanoid | membrane phospholipid |
| Chenodeoxycholic acid | carboxylic acid | bile acid | cholesterol |
| Cholic acid | carboxylic acid | bile acid | cholesterol |
| Docosahexaenoic acid | omega-3-fatty acid | Eicosanoid | alpha linoleic acid |
| Docosapentaenoic acid | omega-3-fatty acid | Eicosanoid | Eicosapentaenoic acid |
| Eicosapentaenoic acid | omega-3-fatty acid | Eicosanoid | alpha linoleic acid |
| Eicosatrienoic acid | omega-3-fatty acid | Eicosanoid | Arachidonic acid |
| Gamma Linolenic acid | omega-6-fatty acid | Eicosanoid | Linoleic acid |
| Glycochenodeoxycholic acid | carboxylic acid | conjugated bile acid | chenodeoxycholic acid |
| Glycocholic acid | carboxylic acid | conjugated bile acid | cholic acid |
| Glycolithocholic acid | carboxylic acid | conjugated bile acid | lithicholic acid |
| Leukotriene B4 | dihydroxy fatty acid | leukotriene | leukotriene A4 |
| Linoleic acid | omega-6-fatty acid | eicosanoid | phospholipid |
| Myristic acid | saturated fatty acid | fatty acid | phospholipid |
| Myristoleic acid | omega-5-fatty acid | unsaturated fatty acid | myristic acid |
| Oleic acid | omega-9-fatty acid | monounsaturated fatty acid | stearic acid |
| Palmitic acid | saturated fatty acid | fatty acid | triglycerides |
| Palmitoleic acid | omega-7-monounsaturated fatty acid | fatty acid | palmitic acid |
| PGE1 | prostaglandin | eicosanoid | PGH1 |
| PGE2 | prostaglandin | eicosanoid | PGH2 |
| Sphingosine 1 Phosphate | lysosphingolipid | sphingolipid | sphingosine |
| Stearic acid | saturated fatty acid | fatty acid | carbohydrates |
| Taurochenodeoxycholic acid | carboxylic acid | conjugated bile acid | chenodeoxycholate |
| Taurocholic acid | carboxylic acid | conjugated bile acid | cholic acid, cholesterol |
| Ursodeoxycholic acid | carboxylic acid | secondary bile acid | deoxycholic acid |
| Ursodeoxycholylglycine | carboxylic acid | conjugated bile salt | ursodeoxycholic acid |
| Ursodeoxycholyltaurine | carboxylic acid | amphiphilic bile acid | ursodeoxycholic acid |

**Table S2. List of top SNP-Metabolite associations, for each possible metabolite.**

| **SNP** | **Chr.** | **Gene** | **Gene Distance**  **(bp)** | **Metabolite** | **Raw *P* value** | **FDR** |
| --- | --- | --- | --- | --- | --- | --- |
| rs16871986 | 7 | *ATXN7L1* | 0 | triHEPE_0650 | 2.10E-07 | 0.028 |
| rs16873183 | 7 | *RAPGEF5* | 0 | triHEPE_0650 | 2.10E-07 | 0.028 |
| rs17146355 | 7 | *RAPGEF5* | 0 | triHEPE_0650 | 2.10E-07 | 0.028 |
| rs1028014 | 8 | *ZMAT4* | 0 | triHEPE_0650 | 3.39E-07 | 0.034 |
| rs11689020 | 2 | *WDR33* | 0 | triHETE_0334 | 4.08E-07 | 0.054 |
| rs17599246 | 2 | *WDR33* | 0 | triHETE_0334 | 4.08E-07 | 0.054 |
| rs6940277 | 6 | *PACRG* | 0 | triHETE_0334 | 4.08E-07 | 0.054 |
| rs11580295 | 1 | *HAO2* | -70000 | Eicosatrienoic_acid | 1.74E-07 | 0.070 |
| rs9522789* | 13 | *LINC000559* | -150000 | LTB4 | 2.51E-07 | 0.10 |
| rs7047072 | 9 | *NAMA* | 40000 | monoHEPE_0910 | 2.61E-07 | 0.10 |
| rs10051603 | 5 | *COX7C* | 150000 | triHEPE_0463 | 4.75E-07 | 0.11 |
| rs7595381 | 2 | *WDR33* | 0 | triHEPE_0463 | 5.25E-07 | 0.11 |
| rs10157096 | 1 | *IGSF21* | 0 | triHEPE_0650 | 1.63E-06 | 0.11 |
| rs7514110 | 1 | *IGSF21* | 0 | triHEPE_0650 | 1.63E-06 | 0.11 |
| rs12498466 | 4 | *ATP10D* | 0 | Adrenic_acid | 3.30E-07 | 0.13 |
| rs6476804 | 9 | *GLIS3* | 0 | Myristoleic_acid | 1.04E-06 | 0.14 |
| rs7147228* | 14 | *CDKL1* | 0 | Myristoleic_acid | 4.38E-07 | 0.14 |
| rs7861853 | 9 | *GLIS3* | 0 | Myristoleic_acid | 1.04E-06 | 0.14 |
| rs7228930 | 18 | *CBLN2* | 20000 | LTB4 | 7.35E-07 | 0.15 |
| rs759582* | 12 | *CMAS* | 10000 | Myristoleic_acid | 1.57E-06 | 0.16 |
| rs11580295 | 1 | *HAO2* | -70000 | Docosahexaenoic_acid | 4.46E-07 | 0.18 |
| rs1113353 | 2 | *LRP1B* | 0 | monoHETE_0984 | 1.80E-06 | 0.18 |
| rs4444359 | 16 | *LOC101927131* | 0 | monoHETE_0984 | 1.80E-06 | 0.18 |
| rs6498196 | 16 | *LOC400499* | 0 | monoHETE_0984 | 1.80E-06 | 0.18 |
| rs7201423* | 16 | *LOC101927131* | 0 | monoHETE_0984 | 1.34E-06 | 0.18 |
| rs2017180 | 17 | *ASIC2* | 45000 | LTB4 | 2.70E-06 | 0.18 |
| rs4794993 | 17 | *ASIC2* | 45000 | LTB4 | 2.70E-06 | 0.18 |
| rs7359592 | 17 | *ASIC2* | 45000 | LTB4 | 2.70E-06 | 0.18 |
| rs8021892 | 14 | *TRA* | 0 | LTB4 | 2.65E-06 | 0.18 |
| rs1552316 | 8 | *SNTG1* | 0 | triHEPE_0463 | 1.98E-06 | 0.20 |
| rs9643689 | 8 | *SNTG1* | 0 | triHEPE_0463 | 1.98E-06 | 0.20 |

Associations ordered by FDR, where FDR < 0.20 was considered top associations. Nearest gene to the SNP is indicated, with approximate distance to that gene (bps). *Indicates SNPs identified for prediction of asthma control phenotype in the Bayesian Network analysis.

**Table S3. Top 20 mRNA-metabolite associations.**

| **Probe ID** | **Gene Symbol** | **Chr.** | ***P* value** | **Metabolite** | **Adjusted *P* value** |
| --- | --- | --- | --- | --- | --- |
| 4250196 | *DSCR1L1* | 6 | 7.04E-08 | Tauroursodeoxycholic_acid | 0.079 |
| 1400373 | *SLA* | 8 | 7.16E-08 | HDHA_1074 | 0.079 |
| 2340008 | *PDZD8* | 10 | 1.15E-07 | HDHA_1074 | 0.079 |
| 4070259 | *GNA12* | 7 | 1.54E-07 | HDHA_1074 | 0.079 |
| 2360348 | *CMPK2* | 2 | 1.61E-07 | Docosatrienoic_acid | 0.079 |
| 6510170 | *IFIT3* | 10 | 5.58E-07 | Docosatrienoic_acid | 0.202 |
| 6280543 | *OASL* | 12 | 5.80E-07 | Docosatrienoic_acid | 0.202 |
| 7040035 | *OAS1* | 12 | 8.17E-07 | Docosatrienoic_acid | 0.225 |
| 2600747 | *IFIT2* | 10 | 8.30E-07 | Docosatrienoic_acid | 0.225 |
| 4570441 | *IFIH1* | 2 | 9.51E-07 | Docosatrienoic_acid | 0.230 |
| 4210524 | *RND3* | 2 | 1.22E-06 | Tauroursodeoxycholic_acid | 0.230 |
| 3850543 | *FLJ31945* | 13 | 1.36E-06 | Taurochenodeoxycholic_acid | 0.230 |
| 6330132 | *ISG20* | 15 | 1.37E-06 | Docosatrienoic_acid | 0.230 |
| 3890609 | *PLSCR1* | 3 | 1.46E-06 | Docosatrienoic_acid | 0.230 |
| 1690039 | *PRKCE* | 2 | 1.51E-06 | HDHA_1074 | 0.230 |
| 4610131 | *SPRR3* | 1 | 1.62E-06 | Tauroursodeoxycholic_acid | 0.230 |
| 610332 | *CHN1* | 2 | 1.63E-06 | Glycocholic_acid | 0.230 |
| 2230343 | *RABIF* | 1 | 1.88E-06 | HDHA_1074 | 0.230 |
| 3450180 | *OAS1* | 12 | 2.11E-06 | Docosatrienoic_acid | 0.230 |
| 3610475 | *LOC402057* | 22 | 2.15E-06 | Docosapentaenoic_acid | 0.230 |

**Table S4. Top Metabolite-methylation Associations.**

| **Methylation Site** | **CpG type** | **Chr** | **Gene(s) (distance, bps)** | **Metabolite** | ***P* value** | **Adjusted  *P* value** |
| --- | --- | --- | --- | --- | --- | --- |
| cg14300730 | II | 2 | *SULT1C2P1, SULT1C4* (+20k) | Tauroursodeoxycholic_acid | 6.47E-08 | 4.14E-06 |
| cg21167159 | II | 11 | *HOTS* (+10k) | Tauroursodeoxycholic_acid | 1.68E-07 | 1.08E-05 |
| cg13232900 | II | 16 | *ADAT1, KARS* (+10k) | Ursodeoxycholic_acid | 2.84E-07 | 1.82E-05 |
| cg00764307 | II | 14 | *ASB2, FAM181A* (+20k) | diHEPE_0753 | 4.60E-07 | 2.94E-05 |
| cg07872854 | II | 19 | *ACSBG2, MLLT1* (+20k) | Chenodeoxycholic_acid | 5.99E-07 | 3.84E-05 |
| cg16274199 | I | 5 | *SRFBP1* (+30k) | Ursodeoxycholic_acid | 6.60E-07 | 4.22E-05 |
| cg20437495 | I | 19 | *AP1M1, CIB3, FAM32A, HSH2D* (+20k) | diHEPE_0753 | 6.69E-07 | 4.28E-05 |
| cg00431835 | II | 16 | *FOXC2* (+60k) | triHEPE_0463 | 9.12E-07 | 5.84E-05 |
| cg20080320 | II | 15 | *HERC2, OCA2* (+20k) | Chenodeoxycholic_acid | 1.02E-06 | 6.50E-05 |
| cg23633635 | I | 17 | *PLSCR3* | triHEPE_0463 | 1.06E-06 | 6.80E-05 |
| cg15303793 | II | 2 | *ICOS, PARD3B* (+90k) | diHETE_0548 | 1.07E-06 | 6.88E-05 |
| cg22510337 | I | 19 | *ZNF417, ZNF587, ZNF814* (+20k) | monoHETE_0809 | 1.08E-06 | 6.93E-05 |
| cg21380380 | II | 7 | *GLCCI1* (+10k) | Taurocholic_acid | 1.08E-06 | 6.94E-05 |
| cg09917029 | II | 4 | *DHX15* (+70k) | monoHETE_0809 | 1.32E-06 | 8.46E-05 |
| cg05483125 | II | 16 | *CHST5, CHST6* (+20k) | Palmitoleic_acid | 1.36E-06 | 8.73E-05 |
| cg05533340 | II | 11 | *HTATIP2, PRMT3* (+40k) | diHETE_0548 | 1.48E-06 | 9.46E-05 |
| cg11189139 | I | 20 | *PDRG1, TTLL9* (+10k) | triHETE_0334 | 1.94E-06 | 0.000124 |
| cg03269218 | I | 10 | *ACSM6, PDLIM1* (+10k) | Docosapentaenoic_acid | 2.76E-06 | 0.000176 |
| cg06934988 | I | 17 | *USP43, WDR16* (+10k) | Docosatrienoic_acid | 3.57E-06 | 0.000228 |

Top two associations per metabolite were considered; for associations that were among the top 50 of all associations by adjusted *P* value. The adjusted p-value was calculated for each metabolite and for each type of CpG sites. Specifically, for each of the 64 metabolites, we tested its association with each of the 135,464 type I CpG sites (346,914 type II CpG sites). We then adjusted p-value for controlling the 135,464 (346,914) tests so that FDR < 0.05.

**Table S5. Candidate Gene Pathways.**

| **Pathway Name** | **Genes** | **Total No. Genes** | **No. Overlapping Genes** | **Unadjusted  *P* Value** | **Adjusted  *P* Value** |
| --- | --- | --- | --- | --- | --- |
| Interferon alpha/beta signaling | *IFIT2, IFIT3, OASL, OAS1, ISG20* | 77 | 5 | 9.67E-11 | 4.74E-09 |
| Interferon Signaling | *IFIT2, IFIT3, OASL, OAS1, ISG20* | 98 | 5 | 3.31E-10 | 8.11E-09 |
| Cytokine Signaling in Immune system | *IFIT2, IFIT3, OASL, OAS1, ISG20* | 193 | 5 | 1.01E-08 | 1.65E-07 |
| Immune System | *IFIT2, IFIT3, IF1H1, OASL, OAS1, ISG20* | 532 | 6 | 3.78E-08 | 4.63E-07 |
| Thromboxane A2 receptor signaling | *PRKCE, GNA12* | 54 | 2 | 0.0002 | 0.0016 |
| Interferon gamma signaling | *OASL, OAS1* | 47 | 2 | 0.0002 | 0.0016 |
| Type II interferon signaling (IFNG) | *IFIT2, OAS1* | 79 | 2 | 0.0004 | 0.0006 |
| G Protein Signaling Pathways | *PRKCE, GNA12* | 95 | 2 | 0.0006 | 0.0006 |
| LPA receptor mediated events | *PRKCE, GNA12* | 100 | 2 | 0.0007 | 0.0049 |
| EGF receptor (ErbB1) signaling pathway | *PRKCE, GNA12, SLA1* | 1288 | 3 | 0.0132 | 0.016 |
| Arf6 downstream pathway | *PRKCE, GNA12, SLA1* | 1288 | 3 | 0.0132 | 0.016 |
| Insulin Pathway | *PRKCE, GNA12, SLA1* | 1288 | 3 | 0.0132 | 0.016 |

**Table S6. Connectivity Map Query**

| **Rank** | **Compound Name** | **n** | **Enrichment Score** |
| --- | --- | --- | --- |
| 1 | salbutamol | 2 | 1 |
| 2 | metaraminol | 2 | 1 |
| 3 | 0173570-0000 | 4 | 0.99 |
| 4 | AG-013608 | 4 | 0.95 |
| 5 | tanespimycin | 12 | 0.73 |
| 6 | colforsin | 2 | 1.00 |
| 7 | alprostadil | 3 | 0.99 |
| 8 | CP-320650-01 | 4 | 0.94 |
| 9 | 16-phenyltetranorprostaglandin E2 | 2 | 0.99 |
| 10 | dinoprostone | 2 | 0.99 |
| 11 | 11-deoxy-16,16-dimethylprostaglandin E2 | 2 | 0.99 |
| 12 | mianserin | 2 | -1.00 |
| 13 | 16,16-dimethylprostaglandin E2 | 2 | 0.99 |
| 14 | CP-319743 | 2 | 0.99 |
| 15 | alexidine | 2 | 0.99 |
| 16 | Prestwick-665 | 2 | 0.99 |
| 17 | orphenadrine | 2 | -0.99 |
| 18 | moxisylyte | 2 | -0.99 |
| 19 | geldanamycin | 2 | 0.99 |
| 20 | mebendazole | 2 | 0.98 |

Gene expression signatures were determined for salbutamol and used to query the connectivity map. The results show the rank, compound name, the number of independent experiments performed, and enrichment scores for the top-ranked 20 compounds (*P* < 5.0E-05 for all compounds shown).

**Table S7. S1P Pathway Genes Enriched in PGE2 Gene Expression Signature.**

| **Probe ID** | **Gene Symbol** | **Score** | **Amplitude** |
| --- | --- | --- | --- |
| 203980_at | *FABP4* | 0.006 | 1.59 |
| 39248_at | *AQP3* | 0.015 | 1.35 |
| 201041_s_at | *DUSP1* | 0.239 | 0.8 |
| 201869_s_at | *TBL1X* | 0.276 | 0.74 |
| 212236_x_at | *KRT17* | 0.284 | 0.74 |
| 205681_at | *BCL2A1* | 0.305 | 0.7 |
| 207630_s_at | *CREM* | 0.321 | 0.69 |
| 217400_at | *PCNA* | 0.336 | 0.68 |
| 214508_x_at | *CREM* | 0.351 | 0.66 |
| 206405_x_at | *USP6* | 0.023 | 0.62 |
| 201867_s_at | *TBL1X* | 0.399 | 0.58 |
| 213400_s_at | *TBL1X* | 0.429 | 0.57 |
| 201868_s_at | *TBL1X* | 0.442 | 0.54 |
| 204855_at | *SERPINB 5* | 0.478 | 0.51 |
| 209967_s_at | *CREM* | 0.478 | 0.47 |
| 207351_s_at | *SH2D2A* | 0.491 | 0.45 |
| 209200_at | *MEF2C* | 0.051 | 0.44 |
| 211370_s_at | *MAP2K5* | 0.055 | 0.43 |
| 210538_s_at | *BIRC3* | 0.066 | 0.38 |
| 206501_x_at | *ETV1* | 0.51 | 0.37 |
| 201598_s_at | *INPPL1* | 0.068 | 0.37 |
| 219278_at | *MAP3K6* | 0.528 | 0.32 |
| 207658_s_at | *FOXG1* | 0.057 | 0.27 |
| 38521_at | *CD22* | 0.053 | 0.22 |
| 211143_x_at | *NR4A1* | 0.032 | 0.17 |
| 219610_at | *ARHGEF28* | 0.046 | 0.12 |
| 221428_s_at | *TBL1XR1* | 0.04 | 0.08 |
| 203904_x_at | *CD82* | 0.454 | 0 |
| 205253_at | *PBX1* | 0.319 | 0 |
| 205396_at | *SMAD3* | -0.292 | -0.01 |
| 209305_s_at | *GADD45B* | -0.299 | -0.05 |
| 218158_s_at | *APPL1* | -0.326 | -0.12 |
| 216262_s_at | *TGIF2* | -0.336 | -0.14 |
| 209640_at | *PML* | 0.123 | -0.14 |
| 210764_s_at | *CYR61* | -0.332 | -0.16 |
| 213906_at | *MYBL1* | -0.327 | -0.16 |
| 211013_x_at | *PML* | -0.328 | -0.19 |
| 214021_x_at | *ITGB5* | -0.32 | -0.2 |
| 201466_s_at | *JUN* | -0.316 | -0.2 |
| 202609_at | *EPS8* | -0.305 | -0.22 |
| 221039_s_at | *ASAP1* | -0.29 | -0.23 |
| 204004_at | *PAWR* | -0.276 | -0.25 |
| 209457_at | *DUSP5* | -0.267 | -0.25 |
| 201902_s_at | *YY1* | 0.06 | -0.26 |
| 206170_at | *ADRB2* | -0.264 | -0.29 |
| 204790_at | *SMAD7* | -0.236 | -0.32 |
| 213295_at | *CYLD* | -0.214 | -0.37 |
| 205447_s_at | *MAP3K12* | -0.112 | -0.54 |
| 201289_at | *CYR61* | -0.097 | -0.56 |
| 202628_s_at | *SERPINE1* | -0.076 | -0.74 |
| 209101_at | *CTGF* | -0.065 | -0.75 |
| 205448_s_at | *MAP3K12* | -0.049 | -0.79 |
| 217028_at | *CXCR4* | -0.039 | -0.86 |
| 211160_x_at | *ACTN1* | -0.011 | -0.94 |
| 202627_s_at | *SERPINE1* | 0.001 | -0.94 |

Gene expression signatures were determined for ‘salbutamol’ (albuterol) and used to query the connectivity map. As shown in **Table S6**, ‘dinoprostone’ (PGE2) was highly positively correlated with albuterol, indicating similar effects on the gene expression signature of albuterol. The results show probes (genes) from the albuterol gene expression signature that were modulated by PGE2 and annotated to the S1P pathway. The shaded rows indicate probes that are -0.4 to +0.4 (1.5-fold) up- or down-regulated in response to PGE2 treatment.

**Table S8. Chart showing if a subject had genotype data, metabolite data, expression data, or methylation data.**

| **Subject ID** | **Genotype data** | **Metabolite data** | **Expression data** | **Methylation data** | **Having all 4 types of data** |
| --- | --- | --- | --- | --- | --- |
| 506005004 | X | X | X | X | Yes |
| 506005011 | X | X | X | X | Yes |
| 506005033 | X | X | X | X | Yes |
| 506005111 | X | X | X | X | Yes |
| 506005116 | X | X | X | X | Yes |
| 506005142 | X | outlier |  | X |  |
| 506005146 | X | X | excluded | X |  |
| 506005202 | X | X | X | X | Yes |
| 506005203 | X | outlier |  |  |  |
| 506005206 | X | X | X | X | Yes |
| 506005208 | X | X | X |  |  |
| 506005209 | X | X | X |  |  |
| 506005221 | X | X | X |  |  |
| 506005301 | X | X | X |  |  |
| 506005304 | X | X | X |  |  |
| 506005320 | X | outlier |  |  |  |
| 506005328 | X | outlier |  | X |  |
| 506005339 | X | X | X | X | Yes |
| 506005340 | X | X | excluded | X |  |
| 506005344 | X | X | X | X | Yes |

“X” indicates having the data; Whitespace indicates no data; “outlier” indicates outliers that were included in subsequent analyses when appropriate; “excluded” indicates outliers that were excluded from subsequent analysis.
